# A protocol update for the Selenium Treatment and Chagasic Cardiomyopathy (STCC) trial

**DOI:** 10.1186/s13063-018-2889-8

**Published:** 2018-09-19

**Authors:** Marcelo Teixeira Holanda, Mauro Felippe Felix Mediano, Alejandro Marcel Hasslocher-Moreno, Sérgio Salles Xavier, Roberto Magalhães Saraiva, Andrea Silvestre Sousa, Erica Rodrigues Maciel, Fernanda Martins Carneiro, Paula Simplicio da Silva, Luiz Henrique Conde Sangenis, Henrique Horta Veloso, Claudia Santos de Aguiar Cardoso, Maria da Gloria Bonecini-Almeida, Andreia Lamoglia Souza, Eric Henrique Roma, Marcos José Azevedo, Fernanda Sant’Ana Pereira-Silva, Luis Otavio Pimentel, Marcelo Oliveira Mendes, Luciana Ribeiro Garzoni, Beatriz M. S. Gonzaga, Anna Cristina Calçada Carvalho, Pedro Emmanuel Alvarenga Americano Brasil, Gilberto Marcelo Sperandio da Silva, Tania Cremonini Araújo-Jorge

**Affiliations:** 1 Evandro Chagas National Institute of Infectious Disease, Oswaldo Cruz Foundation, Rio de Janeiro, Brazil; 2 Laboratory of Innovations in Therapies, Education and Bioproducts, Oswaldo Cruz Institute, Oswaldo Cruz Foundation, Rio de Janeiro, Brazil

**Keywords:** Chagas cardiomyopathy, Chagas disease, Clinical trial, Selenium, *Trypanosoma cruzi*

## Abstract

**Abstract:**

Several studies evaluating clinical forms of chronic Chagas disease show that about one-third of patients present cardiac involvement. Heart failure, sudden death and cardioembolic stroke are the main mechanisms of death in Chagas heart disease. The impact of specific etiologic treatment on the prognosis of patients with chronic Chagas heart disease is very limited regardless of the presence or absence of heart failure. Patients with symptomatic Chagas heart disease present serum selenium (Se) levels lower than patients without Chagas heart disease. Moreover, Se supplementation in animal models showed promising results. The aim of this trial is to estimate the effect of Se treatment on prevention of heart disease progression in patients with Chagas cardiomyopathy. However, we had to introduce some protocol modifications in order to keep trial feasibility, as follows: the primary outcome was restricted to left ventricular ejection fraction as a continuous variable, excluding disease progression; the follow-up period was decreased from 5 years to 1 year, an adjustment that might increase the participation rate of our study; the superior age limit was increased from 65 to 75 years; and diabetes mellitus was no longer considered an exclusion criterion. All of these protocol modifications were extensively debated by the research team enrolled in the design, recruitment and conduction of the clinical trial to guarantee a high scientific quality.

**Trial registration:**

Clinical Trials.gov, NCT00875173. Registered on 20 October 2008.

## Background

Chagas disease is an endemic zoonosis caused by the protozoan parasite *Trypanosoma cruzi*. Currently, about 6–7 million people are infected with *T. cruzi* worldwide [1]. In the Americas about 30,000 new cases/year are registered, with 14,000 deaths/year [2]. Chagas cardiomyopathy is a long-term evolving inflammatory disease that affects around one-third of chronically infected patients and is responsible for high morbidity and mortality [3–5]. In 2014, we proposed the Selenium Treatment and Chagasic Cardiomyopathy (STCC) study aiming to show superiority of treatment with a daily fixed dose of 100 μg of sodium selenite for 1 year during a 5-year follow-up thereafter [6]. The original protocol included left ventricular ejection fraction (LVEF) as a continuous variable and disease progression as the primary outcome. However, the original published study protocol had to be modified due to some practical and theoretical difficulties during the study follow-up. The present article describes the protocol modifications in the STCC study and the rationale that supports such modifications.

### Protocol update

The STCC trial is a superiority, double-blind, placebo-controlled, randomized clinical trial aiming to investigate the efficacy of selenium supplementation in patients with Chagas cardiomyopathy. The full description of the STCC trial was published previously [6]. In brief, patients with a positive serological diagnosis of Chagas disease (confirmed by two different serological tests), aged 18–65 years and presenting mild or moderate left ventricular systolic dysfunction should be included in the study. The intervention protocol comprised 100 μg of sodium selenite once per day for 365 consecutive days compared to placebo. The primary outcomes of the original protocol were: the trajectories of the LVEF as a continuous variable; and disease progression, defined as a 10% decrease in LVEF, death attributable to Chagas heart disease or hospital admission due to arrhythmia, stroke or heart failure. STCC trial recruitment started in 2014 and aimed to include 130 patients to be followed for 5 years. The sample size was calculated based on the rate of disease progression described in Virgem da Lapa [7], which is reproduced in our hospital urban cohort [8] and obviously required a larger sample size.

Study recruitment began in 2014 and since then several pragmatic situations have made the implementation of the original protocol difficult, especially regarding the recruitment rate that was lower than previously predicted. Two main reasons may explain the low recruitment rate observed in the STCC trial: patients with Chagas disease are aging and developing several comorbidities [9] that were a priori determined as the STCC trial’s exclusion criteria, such as diabetes, hypertension and dyslipidemia. The mean age of our cohort moved from 45.9 to 60.5 years in the last 15 years with a current prevalence of several comorbidities ranging from 30 to 60%, thus decreasing the number of patients potentially eligible for the STCC study. In addition, since the original study protocol planned a long-term follow-up (5 years) with frequent outcome evaluations and clinical assessments (some of them once a month), few patients agreed to participate in the clinical trial, mainly due to logistical difficulties in visiting the study center frequently. In this context, some protocol modifications were warranted to maintain study viability without compromising the study design and the potential scientific knowledge generated by its results.

We performed four main modifications of the original protocol. The first was the primary outcome restriction to LVEF as a continuous variable, excluding disease progression. LVEF is a surrogate outcome frequently used in cardiology trials [10] that provides very useful information about the patient’s clinical status and had already been included as a primary outcome in the original protocol. Moreover, LVEF is the single most powerful mortality predictor in Chagas heart disease [11]. Therefore, the sample size was recalculated based on the study conducted by Witte et al. [12] that evaluated the influence of micronutrient supplementation in patients with heart failure and observed an improvement in LVEF of 5.3 ± 6.2%. Considering a beta error of 20% and an alpha error of 5% and anticipating for 30% of losses to follow-up, 62 patients would be necessary (31 in each group) for the study.

The second modification was a decrease in the follow-up period from 5 years to 1 year, an adjustment that might increase the participation rate of our study. Nonetheless, considering that selenium supplementation was provided only during the first year of follow-up, it seems reasonable to evaluate the selenium effects just during the period in which selenium was provided to participants, when the most benefits were expected. In addition, a 1-year follow-up appeared to be a sufficient time to detect LVEF changes [12], given that survival analysis for disease progression would no longer be evaluated in the STCC trial.

The last two modifications were made in the inclusion/exclusion criteria to improve the recruitment rates: the superior age limit was increased from 65 to 75 years and diabetes mellitus was no longer considered an exclusion criterion. All of these protocol modifications were extensively debated by the research team enrolled in the design, recruitment and conduction of the clinical trial to guarantee a high scientific quality. The Evandro Chagas National Institute of Infectious Disease Institutional Ethics Committee approved all of the proposed protocol modifications.

## References

[1] World Health Organization. Chagas disease (American trypanosomiasis): key facts. 2018. www.who.int/news-room/fact-sheets/detail/chagas-disease-(american-trypanosomiasis). Accessed 1 Feb 2018.

[2] Pan American Health Organization. El futuro de la lucha contra el Chagas: proteger los logros, detectar y atender más casos, e interrumpir la transmisión de madre a hijo. 2018. https://www.paho.org/hq/index.php?option=com_content&view=article&id=14313:el-futuro-de-la-lucha-contra-el-chagas-proteger-los-logros-detectar-y-atender-mas-casos-e-interrumpir-la-transmision-de-madre-a-hijo&Itemid=1926&lang=fr. Accessed 1 July 2018.

[3] Martins-Melo FR, Ramos AN, Alencar CH, Heukelbach J (2012). Mortality due to Chagas disease in Brazil from 1979 to 2009: trends and regional differences. J Infect Dev Ctries.

[4] Nóbrega AA, Araújo WN, Vasconcelos AM (2014). Mortality due to Chagas disease in Brazil according to a specific cause. Am J Trop Med Hyg.

[5] Nadruz Wilson, Gioli-Pereira Luciana, Bernardez-Pereira Sabrina, Marcondes-Braga Fabiana G, Fernandes-Silva Miguel M, Silvestre Odilson M, Sposito Andrei C, Ribeiro Antonio L, Bacal Fernando, Fernandes Fabio, Krieger Jose E, Mansur Alfredo J, Pereira Alexandre C (2018). Temporal trends in the contribution of Chagas cardiomyopathy to mortality among patients with heart failure. Heart.

[6] Brasil PEAA, Souza AP, Hasslocher-Moreno AM, Xavier SS, Lambert Passos SR, Moreira MFR, Oliveira MS, Sperandio-da-Silva GM, Saraiva RM, Cardoso CSA, Sousa AS, Mediano MF, Bonecini de Almeida Mda G, da Cruz Moreira O, Britto C, de Araújo-Jorge TC (2014). Selenium Treatment and Chagasic Cardiopathy (STCC): study protocol for a double-blind randomized controlled trial. Trials.

[7] Borges-Pereira J, Xavier SS, Pirmez C, Coura JR (1998). Chagas’ disease in Virgem da Lapa, Minas Gerais, Brazil. IV. Clinical and epidemiological aspects of left ventricle aneurysm. Rev Soc Bras Med Trop.

[8] Salles G, Xavier S, Sousa A, Hasslocher-Moreno A, Cardoso C (2003). Prognostic value of QT interval parameters for mortality risk stratification in Chagas’ disease: results of a long-term follow-up study. Circulation.

[9] Alves RM, Thomaz RP, Almeida EA, Wanderley Jda S, Guariento ME (2009). Chagas’ disease and ageing: the coexistence of other chronic diseases with Chagas’ disease in elderly patients. Rev Soc Bras Med Trop.

[10] Katsi V, Georgiopoulos G, Laina A, Koutli E, Parissis J, Tsioufis C, Nihoyannopoulos P, Tousoulis D (2017). Left ventricular ejection fraction as therapeutic target: is it the ideal marker?. Heart Fail Rev.

[11] Rassi A, Rassi A, Rassi SG (2007). Predictors of mortality in chronic Chagas disease: a systematic review of observational studies. Circulation.

[12] Witte KK, Nikitin NP, Parker AC, von Haehling S, Volk HD, Anker SD, Clark AL, Cleland JG (2005). The effect of micronutrient supplementation on quality-of-life and left ventricular function in elderly patients with chronic heart failure. Eur Heart J.

